# Targeting glycogen metabolism: a novel strategy to inhibit cancer cell growth?

**DOI:** 10.18632/oncotarget.841

**Published:** 2013-01-27

**Authors:** Elena Favaro, Adrian L. Harris

**Affiliations:** Molecular Oncology Laboratories, Weatherall Institute of Molecular Medicine, University of Oxford, John Radcliffe Hospital, Oxford, UK; Molecular Oncology Laboratories, Weatherall Institute of Molecular Medicine, University of Oxford, John Radcliffe Hospital, Oxford, UK

Metabolic reprogramming in cancer cells provides energy and important metabolites required to sustain tumor proliferation [1]. In our recent paper in *Cell Metabolism,* we demonstrate that glycogen mobilization is a common feature of cancer cell metabolism, and may therefore represent a novel anticancer therapeutic target [2]. Glycogen primarily acts as an intracellular storage of glucose and fulfills important roles (in both non-malignant and cancer cells) under conditions of oxygen and nutrient deprivation. Glycogen phosphorylase is the main enzyme that catalyzes the release of glucose from glycogen. Interestingly, we demonstrated that the liver isoform of glycogen phosphorylase, PYGL, is upregulated in hypoxia, and is required for glycogen breakdown in both tumor xenografts and in cancer cell lines. PYGL depletion leads to glycogen accumulation, impaired redox balance, and a reduction in proliferation due to p53-dependent induction of senescence. Furthermore, all of these phenotypes are more pronounced in hypoxia [2].

The preferential shuttling of glucose through glycogen is intriguing because it occurs even in conditions when extracellular glucose is abundant. Indeed, this phenomenon (referred to as ‘glycogen shunt’) has been observed for a number of other (non-cancer) cell types [3]. This suggests that glycogen performs more complex roles rather than solely acting as an inert intracellular glucose store. Consistent with this idea, we demonstrate that glycogen breakdown is required for the optimal functioning of the pentose phosphate pathway (PPP). This channeling of glucose through the PPP generates nucleotides required for sustained proliferation, as well as reduced nicotinamide adenine dinucleotide phosphate (NADPH), which is an important reducing agent for nucleotide, amino acid and lipid synthesis, and also for ROS scavenging [4]. Our ongoing studies are investigating which other metabolic and biosynthetic pathways are also affected by impaired glycogen mobilization.

So how does glycogen exert these effects on cell metabolism and growth? We envisage two possible scenarios, with PYGL acting as an intracellular glycogen ‘sculptor’ in each case. One possibility is that the precise subcellular localization of glucose release from glycogen could favor its preferential channeling into specific metabolic pathways. Indeed, intracellular compartmentalization and trafficking of glycogen (through the glycogen-binding protein, genethonin-1) has previously been demonstrated [5]. Another possibility is that glycogen performs important signaling roles within cells. For example, AMP-activated protein kinase (AMPK), which is an important regulator of cellular energy homeostasis, is directly inhibited by highly branched glycogen granules [6].

Of clinical significance, our findings implicate glycogen metabolizing enzymes, and PYGL in particular, as promising possible targets for cancer treatment. Indeed, some of these treatments may already exist, as PYGL inhibitors are already in development for the treatment of type 2 diabetes. Although there are no data available in humans, these agents are unlikely to be toxic to most cells because patients affected by Hers’ disease (an inherited glycogen storage disorder caused by deficiency of PYGL) are largely asymptomatic. Furthermore, based on our observations, a number of combination therapies could also be considered. Firstly, because of the enrichment of lysosomes in PYGL-depleted cells, a potentially lethal, and highly specific, drug combination could be predicted with PYGL inhibitors combined with lysosome permeabilizing drugs, such as siramesine [7]. Additionally, given the increased reliance of cancer cells on glycogen metabolism in hypoxia, the combination of PYGL inhibition with antiangiogenic drugs (e.g. bevacizumab) should also be investigated.
